# The Role of Weight-Bearing Computed Tomography in the Assessment and Management of Charcot Foot Deformity: A Narrative Review

**DOI:** 10.3390/medicina62010117

**Published:** 2026-01-06

**Authors:** Nah Yon Kim, Young Yi

**Affiliations:** 1 Department of Orthopedic Surgery, Ewha University Mokdong Hospital, Seoul 07985, Republic of Korea; 2 Department of Orthopaedic Surgery, Ewha Womans University College of Medicine, Seoul 03760, Republic of Korea; 3 Ewha Mokdong MSK Data Center for Sports & Rehabilitation, Seoul 07985, Republic of Korea

**Keywords:** Charcot foot, Charcot neuro-osteoarthropathy, diabetic foot, weight-bearing CT, 3D imaging, foot deformity, surgical reconstruction, biomechanics

## Abstract

Charcot neuro-osteoarthropathy (CNO) is a devastating complication of peripheral neuropathy, characterized by progressive bone and joint destruction that leads to severe foot deformity, ulceration, and a high risk of amputation. The management of CNO is predicated on an accurate understanding of its biomechanical instability, yet conventional imaging modalities like non-weight-bearing computed tomography (CT) and magnetic resonance imaging (MRI) fail to capture the true, load-dependent nature of the deformity. This review elucidates the paradigm shift facilitated by weight-bearing computed tomography (WBCT) in the diagnosis and management of CNO. A comprehensive narrative review of the literature was conducted to synthesize the pathophysiology of CNO, the limitations of conventional imaging, and the technological principles, clinical applications, and future directions of WBCT in CNO management. The review integrates findings on CNO pathophysiology, radiological assessment, and the debate surrounding weight-bearing protocols in conservative management. WBCT provides a three-dimensional, functional assessment of the Charcot foot under true physiological load, overcoming the critical limitations of non-weight-bearing imaging. It reveals the full extent of osseous collapse, unmasking hidden instabilities and enabling the use of novel quantitative 3D metrics for deformity characterization and risk stratification. Clinically, WBCT enhances the entire management pathway, from improving early diagnostic accuracy and informing surgical strategy with patient-specific instrumentation to enabling objective postoperative evaluation of reconstructive outcomes. WBCT is a promising technology that redefines the assessment of CNO from a static, morphological description to a dynamic, quantitative biomechanical analysis. Its integration into clinical practice offers the potential to improve diagnostic precision, optimize surgical planning, and ultimately enhance patient outcomes. The future synergy of WBCT with artificial intelligence holds promise for further advancing patient care, moving towards a predictive and prescriptive model for managing this complex condition.

## 1. Introduction: The Clinical Challenge of Charcot Neuro-Osteoarthropathy

Charcot neuro-osteoarthropathy (CNO) is a progressive and destructive condition of the bones, joints, and soft tissues of the foot and ankle, occurring most commonly as a severe complication of diabetes mellitus [1]. It represents a profound clinical challenge, defined by a catastrophic biomechanical collapse that can lead to severe disfiguring deformity, chronic ulceration, infection, and ultimately, major lower-limb amputation [1]. The management of this condition is critically dependent on an accurate assessment of the foot’s structural integrity. However, a fundamental paradox has long existed in its clinical care: CNO is a load-dependent disease, yet for decades, clinicians have relied on imaging modalities that assess the foot in an unloaded state.

This review explores the evolution of diagnostic imaging for CNO and posits that the advent of weight-bearing computed tomography (WBCT) represents a paradigm shift, offering for the first time a functional, three-dimensional (3D) understanding of these devastating pathologies. Given the heterogeneity of the available literature and the evolving role of WBCT in CNO, a narrative review approach was chosen to provide a comprehensive and clinically oriented synthesis of current evidence.

### 1.1. Pathophysiology and Biomechanical Collapse: A Dual Threat

The pathogenesis of CNO is complex and multifactorial, with two predominant theories—the neuro-traumatic and neuro-vascular—converging to explain the rapid skeletal destruction [1]. The neuro-traumatic theory posits that in an insensate foot, a loss of protective sensation allows for repetitive, unrecognized microtrauma during ambulation. This unabated mechanical stress initiates a cascade of fractures, ligamentous injuries, and joint dislocations that progressively dismantle the foot’s architecture [2]. Concurrently, the neuro-vascular theory suggests that autonomic neuropathy leads to a dysregulation of local blood flow, creating a state of hyperemia through arteriovenous shunting. This increased vascularity promotes the activity of osteoclasts, leading to aggressive bone resorption, osteopenia, and a profound weakening of the skeletal framework [1].

In reality, these two pathways likely coalesce into a vicious cycle: a neurologically weakened and osteopenic skeleton is subjected to relentless mechanical forces, leading to accelerated fragmentation and collapse [1]. This process, clinically staged by the Eichenholtz classification (Stages 0–3), culminates in characteristic deformities such as the midfoot collapse known as a “rocker-bottom foot,” which creates abnormal bony prominences and dramatically increases the risk of ulceration and infection [1,3].

**Figure 1:**
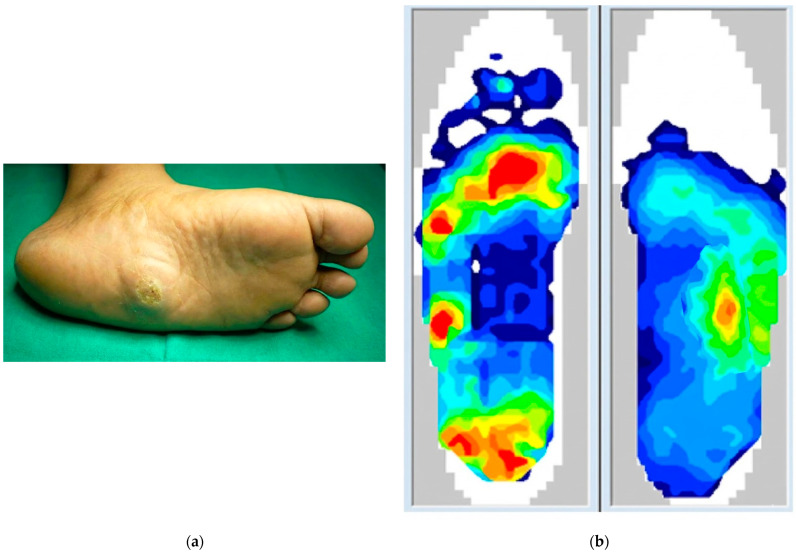

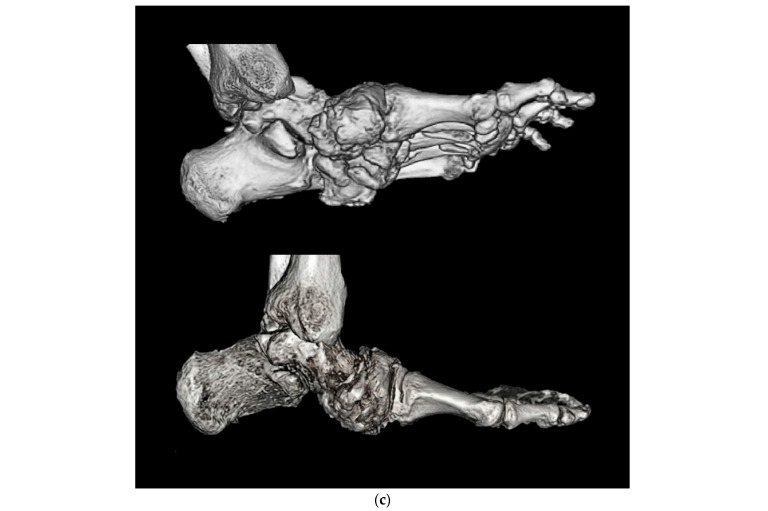
Comprehensive assessment of Charcot Neuro-osteoarthropathy (CNO). (**a**) Clinical photograph showing the classic “rocker-bottom” deformity with a plantar midfoot ulcer, resulting from uncorrected structural collapse. (**b**) Pedobarographic assessment revealing a pathological shift in peak plantar pressure to the midfoot (red zone), confirming the functional impact of the deformity. (**c**) Weight-bearing computed tomography (WBCT) 3D rendering of the same foot. This modality elucidates the underlying osseous destruction and joint subluxation under load that drives the biomechanical failure seen in (**b**) and the clinical outcome seen in (**a**).

This pathophysiology gives rise to a central clinical controversy in the conservative management of active CNO: the role of weight-bearing. The historical standard of care has been strict immobilization in a non-weight-bearing (NWB) total contact cast (TCC), a protocol founded on the theoretical premise of eliminating the traumatic component of the disease [1]. However, this approach is based more on anecdotal evidence and theoretical principles than on high-level clinical data. A systematic review by Prem et al. found no comparative studies between weight-bearing (WB) and NWB protocols and concluded that there is limited evidence to support the current practice of strict NWB [4]. Furthermore, NWB restrictions impose a significant burden on patients, impairing quality of life, increasing dependency, and elevating the risk of complications such as muscle atrophy, thromboembolism, and contralateral limb overload [1]. This long-standing clinical debate is not merely a disagreement over treatment but is a direct consequence of a fundamental diagnostic limitation. Clinicians have been forced to make management decisions based on theory because traditional imaging could not provide empirical data on how a specific patient’s foot deforms under physiological load. The inability to stratify risk or titrate weight-bearing permissions on a patient-specific basis has necessitated a conservative, one-size-fits-all NWB approach. This highlights the critical need for an imaging modality that can precisely quantify the biomechanical effects of weight-bearing, thereby resolving a decades-old clinical and diagnostic impasse. The clinical, functional, and structural consequences of this dual pathophysiology are summarized in Figure 1.

### 1.2. The Diagnostic Dilemma: Differentiating CNO from Osteomyelitis

Compounding the therapeutic challenges of CNO is a significant diagnostic hurdle, particularly in its acute phase (Eichenholtz Stage 0). The initial presentation of CNO is an acutely inflamed foot, characterized by erythema, warmth, and swelling, making it clinically indistinguishable from infectious processes such as cellulitis or osteomyelitis [1]. This diagnostic ambiguity poses a high-stakes clinical problem, as the management strategies for these conditions are diametrically opposed: CNO requires immediate offloading and immobilization, whereas osteomyelitis necessitates antibiotic therapy and potentially urgent surgical debridement [1].

A missed or delayed diagnosis of CNO, which has been reported in a substantial proportion of cases, with rates reported as high as 79% of cases, can have catastrophic consequences, allowing the destructive inflammatory process to continue unchecked and leading to irreversible deformity [5]. The average delay in treatment has been reported to be as long as 29 weeks [6]. This clinical dilemma underscores the imperative for advanced imaging modalities that can accurately differentiate sterile neuro-osteoarthropathy from underlying infection, guiding timely and appropriate intervention to prevent the devastating sequelae of the disease [1].

### 1.3. Overview of Literature Search

This narrative review was based on a literature search conducted using PubMed, Scopus, and Web of Science databases. The search included studies published up to April 2025 and used key terms such as “Charcot neuro-osteoarthropathy,” “weight-bearing computed tomography,” “WBCT,” and “diabetic foot”. Articles were selected based on their relevance to the diagnosis, assessment, and management of Charcot foot deformity, with an emphasis on studies addressing the clinical utility of WBCT.

## 2. Conventional Imaging Modalities: An Incomplete, Non-Functional Perspective

The diagnostic pathway for CNO has traditionally relied on a combination of radiography, magnetic resonance imaging (MRI), and non-weight-bearing computed tomography (CT). While each modality offers unique advantages, they all share a critical, unifying flaw in the context of CNO: they are typically performed with the patient in a non-weight-bearing position, thus failing to capture the true, functional nature of a load-dependent deformity. A comparative overview of these imaging modalities, including their key advantages, limitations, and optimal clinical applications, is summarized in Table 1.

### 2.1. Weight-Bearing Radiography: The First-Line Standard

Weight-bearing radiographs are the fundamental, first-line imaging tool for CNO. They are accessible, cost-effective, and indispensable for confirming a diagnosis in later stages by revealing characteristic findings such as bony consolidation, subchondral bone fragmentation, fractures, and dislocation [1]. Serial radiographs are valuable for monitoring disease progression and assessing gross deformities like midfoot collapse and the development of a rocker-bottom foot [1]. Standard two-dimensional (2D) parameters, including Meary’s angle (talar-first metatarsal angle) and the calcaneal pitch angle, are measured from these images to quantify sagittal plane alignment [1].

Despite their utility, radiographs have inherent limitations. As a 2D projection of a complex 3D structure, radiography is plagued by the superimposition of bones, which can obscure subtle fractures and subluxations, particularly in the anatomically dense midfoot [7]. The accuracy of radiographic measurements is highly susceptible to variations in patient positioning and X-ray beam angulation, leading to poor reproducibility [7]. Most critically, radiography has very low sensitivity for early-stage CNO. In Eichenholtz Stage 0, radiographs are often entirely normal, despite the presence of significant underlying inflammatory bone pathology, leading to false reassurance and diagnostic delay [1].

### 2.2. Magnetic Resonance Imaging (MRI): The Gold Standard for Early and Soft Tissue Diagnosis

MRI is considered the gold standard for the early diagnosis of CNO and for differentiating it from osteomyelitis [1]. Its superior soft tissue and bone marrow contrast allows it to detect the hallmark of Stage 0 CNO—bone marrow edema—long before osseous destruction becomes visible on radiographs [8]. The presence of extensive, periarticular bone marrow edema in multiple bones is highly suggestive of acute CNO, and a negative MRI can effectively rule out the condition [9]. Furthermore, MRI is crucial in the diagnostic dilemma of the “hot, red, swollen foot.” It can identify key imaging features of osteomyelitis, such as sinus tracts extending from a skin ulcer to the bone, soft tissue abscesses, and cortical destruction, which are typically absent in uncomplicated CNO [1]. The “ghost sign,” where bone appears to “dissolve” on T1-weighted images but “reappears” on contrast-enhanced sequences, is another finding suggestive of osteomyelitis [1].

However, the established role of MRI as the “gold standard” creates a diagnostic paradox. While it excels at detecting the presence of acute CNO via inflammation (edema), it is incapable of assessing the direct consequence of that pathology: biomechanical instability under load. Standard MRI is performed with patients in a supine, non-weight-bearing position. This provides an anatomically detailed but functionally artificial depiction of the foot. It cannot assess the degree of joint subluxation, ligamentous instability, or architectural collapse that occurs only when the patient stands and bears weight [7]. This creates a critical disconnect between diagnosis and the functional reality of the disease. A clinician may correctly diagnose Stage 0 CNO based on MRI findings of edema, but the crucial question—“How unstable is this foot when the patient stands on it?”—remains unanswered. Consequently, the treatment plan is based on the presence of inflammation, not on a direct, quantitative measure of the foot’s structural integrity under physiological stress. The conventional imaging pipeline, even when correctly executed, identifies the active inflammatory process but cannot quantify the resultant biomechanical compromise of the skeletal architecture, which is the information most critical for preventing collapse.

### 2.3. Non-Weight-Bearing Computed Tomography (CT): A Deceptive 3D Perspective

Conventional, non-weight-bearing CT offers distinct advantages over both radiography and MRI in specific scenarios. It provides superior visualization of cortical bone detail, making it excellent for delineating complex fracture patterns, identifying bone fragmentation, quantifying bone loss, and assessing for periosteal reactions [1]. This level of detail is particularly valuable for preoperative planning in advanced, quiescent stages of CNO, where surgical reconstruction is contemplated [1].

The critical flaw of conventional CT, however, is identical to that of MRI: it is performed non-weight-bearing. This failure to image the foot under physiological load is a major limitation, as it provides a misrepresentative picture of the joint’s functional state and can lead to a significant underestimation of the deformity’s true severity [10]. Studies that have attempted to simulate axial loads during CT scans have consistently demonstrated that deformities are more pronounced and joint subluxations are more apparent under load, confirming that a non-WBCT provides a deceptively stable view of the Charcot foot [11]. This incomplete assessment can lead to suboptimal treatment planning and a failure to appreciate the true extent of the instability that must be addressed.

**Table 1:** Comparative Analysis of Imaging Modalities for Charcot Neuro-Osteoarthropathy.

Modality	Imaging Principle	Key Advantages of CNO	Key Limitations of CNO	Optimal Clinical Application
Weight-Bearing Radiography	2D projection under load	Accessible; cost-effective; good for assessing gross deformity and serial monitoring of progression [1].	Superimposition of structures; low sensitivity for early (Stage 0) disease; poor reproducibility due to positioning variability [7].	Initial screening and baseline assessment; long-term follow-up of established deformity.
Magnetic Resonance Imaging (MRI)	Multi-planar soft tissue and bone marrow contrast (non-weight-bearing)	Gold standard for Stage 0 diagnosis (detects bone marrow edema); excellent for differentiating CNO from osteomyelitis (detects sinus tracts, abscesses) [1].	Non-weight-bearing, thus underestimates true biomechanical instability and joint subluxation; it can be affected by metallic hardware artifacts [7].	Suspected acute (Stage 0) CNO with negative radiographs; clinical concern for concomitant osteomyelitis.
Conventional (Non-WB) CT	3D cross-sectional bone detail (non-weight-bearing)	Superior cortical bone detail for complex fractures and fragmentation; excellent for quantifying bone loss and preoperative planning in severe, quiescent deformity [1].	Non-weight-bearing, providing a functionally artificial view that misrepresents true alignment and underestimates the severity of collapse [10].	Preoperative planning for major reconstruction in advanced, stable (Eichenholtz Stage 3) deformity, where soft tissue detail is not paramount.
Weight-Bearing CT (WBCT)	3D cross-sectional bone detail under physiological load	Provides true functional assessment of stability; quantifies load-dependent instability and collapse; enables novel 3D biomechanical metrics (e.g., FAO); reveals hidden deformities not seen on non-WB imaging; lower radiation than conventional CT [11].	Higher cost and limited accessibility; inferior soft tissue contrast compared to MRI; interpretation is more complex and time-consuming than 2D imaging; potential for metallic artifacts [7].	Comprehensive assessment of instability at any stage; definitive pre- and postoperative evaluation of alignment for surgical reconstruction.

## 3. The Paradigm Shift: Three-Dimensional Functional Assessment with Weight-Bearing CT

The introduction of weight-bearing computed tomography (WBCT) represents an important advancement in musculoskeletal imaging, directly addressing the fundamental limitations of conventional modalities in the assessment of CNO. By providing a 3D, functional view of the foot and ankle under physiological load, WBCT facilitates a paradigm shift from a static, morphological description of deformity to a dynamic, quantitative biomechanical analysis.

### 3.1. Technological Principles and Advantages over Conventional Imaging

WBCT systems typically utilize cone-beam CT (CBCT) technology, which employs a cone-shaped X-ray beam and a flat-panel detector that rotates around the patient’s extremity while they stand in a natural, weight-bearing position [11]. This process allows for rapid image acquisition that captures the osseous structures under the influence of both body weight and active muscle forces, providing a true functional assessment [7].

The primary advantages of WBCT over conventional imaging are substantial:

- True Functional Imaging: Its ability to provide a 3D, load-bearing assessment is its single most important and defining feature, offering insights into joint stability and alignment that are impossible to obtain with non-WB CT or MRI [10].
- Superior Osseous Detail and High Spatial Resolution: WBCT provides exquisite 3D visualization of bone and joint relationships, free from the superimposition artifacts that plague 2D radiography [11].
- Improved Accuracy and Reproducibility: By standardizing the patient’s position (i.e., standing) and providing 3D data, measurements derived from WBCT are inherently more reproducible and precise than 2D radiographic measurements, which are highly susceptible to rotational and projectional errors [12].
- Lower Radiation Dose: WBCT systems expose patients to a significantly lower dose of ionizing radiation compared to conventional multi-detector CT (MDCT) scanners, with an effective dose that is comparable to a multi-view radiographic series [11].

### 3.2. Biomechanical Insights and Novel Quantitative Metrics

The ability of WBCT to capture the loaded state of the Charcot foot has provided unprecedented biomechanical insights and has spurred the development of novel quantitative metrics to characterize deformity.

Most importantly, WBCT unmasks the true severity of the collapse, which is often underestimated by non-load-bearing studies. A key study comparing WBCT to standard weight-bearing radiographs in patients with midfoot CNO found that while many sagittal plane angles were comparable, the lateral column height was significantly lower when measured on WBCT (mean 12.26 mm) compared to radiographs (mean 19.12 mm) [13]. This finding is of profound clinical significance, as a diminished lateral column height is a direct indicator of midfoot collapse and is strongly correlated with an increased risk of developing lateral foot ulceration. This demonstrates that even weight-bearing radiographs can fail to capture the full extent of the collapse revealed by 3D imaging under load.

This shift from simply describing the shape of the deformity (e.g., “rocker-bottom”) to precisely measuring its instability is a fundamental advance. It allows clinicians to move beyond subjective assessments and base decisions on objective, reproducible data that directly reflect the functional biomechanics of the patient’s foot. This quantification provides the missing link to understanding and predicting the risk of soft tissue complications. Ulceration in CNO is a mechanical event caused by abnormal pressure from underlying bony prominences. By precisely measuring how the foot collapses under load, WBCT provides a direct biomarker for the risk of future skin breakdown. This enables a proactive, rather than reactive, approach to management, where interventions can be planned to prevent ulceration before it occurs.

Furthermore, WBCT has enabled the development and validation of new, semi-automated 3D biometric tools that can quantify complex, multi-planar deformities with high reliability. These include:

- Foot and Ankle Offset (FAO): A 3D metric that describes the relationship between the mechanical axis of the ankle and the weight-bearing tripod of the foot (calcaneus, first metatarsal head, and fifth metatarsal head), providing a robust and reliable measure of hindfoot alignment [12].
- Subfibular and Sinus Tarsi Impingement: WBCT can directly visualize and quantify the degree of bone-on-bone impingement in the hindfoot, a common source of pain and a driver of progressive deformity that is difficult to assess on 2D imaging [10]. In CNO, the degree of subfibular impingement has been shown to correlate with the anatomical location of the primary deformity, providing valuable information for surgical planning [13].
- Rotational Deformity Assessment: WBCT allows for the accurate measurement of rotational malalignment of bones, a critical component of many foot deformities that is completely invisible on standard 2D radiographs [10].

## 4. Clinical Applications of WBCT Throughout the CNO Management Pathway

The unique capabilities of WBCT allow it to be integrated as a powerful tool across the entire spectrum of CNO management, from initial diagnosis and characterization to detailed preoperative planning and objective postoperative assessment.

As summarized in Table 2, WBCT may be particularly useful in specific clinical scenarios, including patients with suspected early-stage CNO and inconclusive conventional imaging, those with established deformity requiring quantitative assessment under load, and patients undergoing preoperative planning for reconstructive surgery. In addition, WBCT can be considered during postoperative follow-up to evaluate alignment and fusion under physiological weight-bearing conditions.

### 4.1. Enhancing Early Diagnosis and Deformity Characterization

In the crucial early stages of CNO (Eichenholtz 0 and 1), where radiographs are often unrevealing, WBCT can play a vital role. While MRI remains superior for detecting the initial inflammatory response of bone marrow edema, WBCT is uniquely suited to assess the biomechanical consequences of that inflammation [10,13]. It can detect latent biomechanical instability—such as subtle joint incongruity or minor subluxations—marking the critical transition from those that are only manifest under physiological load. Identifying this occult, load-dependent instability at an early stage is critical for initiating aggressive offloading therapy to prevent progression to catastrophic collapse.

For established deformities, WBCT provides a comprehensive 3D map of the osseous architecture under load. This allows for a complete understanding of the deformity in all three planes—sagittal, coronal, and axial—which is essential for appreciating the complex, multi-planar nature of CNO collapse that is often oversimplified by 2D imaging [10].

From a clinical perspective, WBCT should be interpreted in conjunction with MRI, as each modality provides distinct and complementary information across different stages of CNO (Table 3).

### 4.2. Preoperative Planning for Surgical Reconstruction

Surgical reconstruction of severe Charcot foot deformity is one of the most challenging procedures in orthopedic surgery, with a goal of creating a stable, plantigrade, and ulcer-free foot [14]. WBCT has become an invaluable tool in the preoperative planning for these complex cases.

By providing a precise visualization of the true, load-bearing alignment, WBCT allows surgeons to more accurately plan corrective osteotomies, determine the optimal location and extent of bone resection and arthrodesis, and select the most appropriate fixation strategy (often a “superconstruct” involving long, rigid internal fixation) required to achieve a stable plantigrade platform [14]. It helps to identify all sites of instability, quantify the remaining bone stock, and pinpoint bony prominences that may lead to ulceration [14]. By accurately modeling the degree of correction required, surgeons can also better anticipate potential challenges with soft tissue tension and wound closure, a common complication in major CNO reconstructions [14].

This detailed preoperative information facilitates a shift in surgical philosophy from “reconstructive” to “restorative.” Instead of simply correcting a visually apparent deformity to achieve a subjectively “plantigrade” foot, surgeons can use WBCT to establish objective, quantitative targets for the reconstruction (e.g., restore lateral column height to a specific value, correct the FAO to within a normal range).

This capability is further enhanced by using patient-specific instrumentation (PSI). The high-resolution 3D data from a preoperative WBCT scan can be used to engineer and 3D-print custom cutting guides and surgical instruments that are perfectly matched to the patient’s unique anatomy [10]. This technology allows for the highly precise execution of the preoperative plan, improving the accuracy of bone cuts, ensuring optimal implant placement, and increasing the reproducibility of the deformity correction [10]. This process transforms the surgery from an art based on visual estimation and freehand techniques to a science based on restoring quantified biomechanical parameters, with significant potential to improve outcomes and reduce the high complication rates associated with CNO surgery [14].

### 4.3. Postoperative Evaluation and Monitoring

Following surgical reconstruction, WBCT serves as the definitive method for evaluating the outcome. While the ultimate goal of surgery is to create a foot that is stable and plantigrade during walking, non-weight-bearing postoperative imaging can be misleading. WBCT provides an objective assessment of the surgical correction underload, confirming that a stable, plantigrade alignment has been achieved and maintained [14]. It is particularly valuable at the transition point when a patient progresses from non-weight-bearing to protected weight-bearing (typically 6–12 weeks post-reconstruction). This timing allows for the objective confirmation of hardware stability and maintenance of plantigrade alignment before full physiological load is applied.

It allows for a precise, 3D evaluation of bony bridging and fusion across arthrodesis sites, which can be challenging to visualize on 2D radiographs due to overlying hardware and complex anatomy [14]. Furthermore, WBCT can identify any residual instability, loss of correction over time, or the development of new areas of collapse at adjacent joints that may not be apparent on non-WB imaging, allowing for timely intervention to prevent failure of the reconstruction [14].

## 5. Limitations and Future Directions

While WBCT represents a significant advance in the management of CNO, it is important to acknowledge its current limitations and to look toward future developments that may further enhance its utility.

### 5.1. Current Challenges and Limitations of WBCT

Several challenges currently temper the widespread adoption of WBCT. The vast amount of 3D data generated requires a significant investment of time for interpretation compared to 2D radiographs and necessitates a new skill set for radiologists and surgeons accustomed to planar imaging [12]. As a cone-beam technology, WBCT images can be susceptible to artifacts, particularly from metallic hardware, which can complicate postoperative evaluation, although modern algorithms are improving this aspect [7]. While its visualization of bone is superb, its ability to assess soft tissues remains inferior to MRI, which is still the modality of choice when soft tissue infection or abscess is a primary concern [1]. In addition, variability related to acquisition protocols, software platforms, and measurement workflows may affect inter-institutional reproducibility of WBCT-derived metrics, underscoring the need for further standardization.

Perhaps the most significant barriers are cost and accessibility. The initial acquisition and maintenance costs of WBCT scanners are substantial. Although studies suggest that the technology can be a financially viable asset in high-volume orthopedic practices, its availability remains limited compared to standard radiography and conventional CT [15].

In this context, while the initial cost of WBCT is higher than radiography, its economic justification lies in the potential prevention of limb-threatening complications. Given that the cost of managing a single CNO-related major amputation and subsequent prosthetic care can exceed the price of advanced diagnostics by several fold, WBCT serves as a cost-effective gatekeeper for high-risk patients.

The economic justification for WBCT must be considered in the context of the extremely high costs associated with managing the complications of CNO, such as failed conservative treatment, chronic ulceration, and amputation, which can be far greater than the cost of advanced diagnostics [16]. Finally, while the radiation dose from WBCT is significantly lower than that of conventional CT, it is still a source of ionizing radiation, unlike MRI [11].

### 5.2. Recent Advances in Weight-Bearing CT for CNO

Recent advances in weight-bearing computed tomography have significantly enhanced its clinical applicability in Charcot neuro-osteoarthropathy. Beyond Charcot neuro-osteoarthropathy, weight-bearing CT has been widely applied in the complex hindfoot and other foot disorders, where it reveals subtalar joint malalignment and osseous contact not evident on non-weight-bearing imaging, and enables three-dimensional assessment of alignment, joint congruity, and load-dependent instability [17,18]. Improvements in cone-beam CT technology have enabled high-resolution, three-dimensional imaging of the foot and ankle under physiological load, with reduced radiation exposure and shorter acquisition times compared with earlier systems [19].

In parallel, there has been growing interest in the development of standardized three-dimensional alignment parameters and quantitative metrics derived from WBCT. These advances allow more precise assessment of hindfoot valgus, midfoot collapse, and talar translation under weight-bearing conditions, addressing key limitations of conventional two-dimensional radiography [10,20].

Furthermore, advances in visualization and analysis software have facilitated more efficient interpretation of complex WBCT datasets, supporting broader clinical adoption [18]. Collectively, these developments position WBCT as an increasingly practical and informative imaging modality in the evaluation and management of CNO.

### 5.3. The Future of CNO Imaging: The Role of Artificial Intelligence (AI)

The future of CNO imaging lies in the synergy between the rich, quantitative data provided by WBCT and the analytical power of artificial intelligence (AI) and machine learning (ML). This combination holds the promise of transforming CNO management from a reactive to a predictive and ultimately prescriptive discipline.

AI and ML algorithms are currently being developed to automate time-consuming image segmentation and measurement tasks from WBCT datasets [21]. This will overcome the current limitation of interpretation complexity and provide clinicians with rapid, objective, and highly reproducible quantitative reports on foot alignment and deformity.

The next frontier is predictive modeling. Quantitative biomechanical data from WBCT are an ideal input for ML models designed to predict clinical outcomes. Future research will likely focus on developing and validating AI models that analyze subtle biomarkers in WBCT scans to predict a patient’s individual risk of deformity progression, ulceration, or failure of a planned surgical construct [21]. At present, however, the use of WBCT-derived metrics for outcome prediction remains largely conceptual and has not yet been validated in prospective clinical studies. This would enable true, personalized risk stratification and allow for preventative interventions tailored to the individual. For example, generative AI models could address the relative scarcity of CNO data by creating large synthetic WBCT datasets that can be used to train and validate these diagnostic and prognostic algorithms more rapidly [21].

Ultimately, this integration may lead to the development of prescriptive imaging. An AI system could analyze a patient’s unique 3D biomechanics from their WBCT scan and then run virtual simulations of various surgical interventions—testing different osteotomy angles, screw placements, or fixation methods. The system could then recommend the optimal surgical plan that maximizes stability, restores normal alignment, and minimizes stress on hardware and soft tissues. This would serve as a powerful decision support tool for surgeons, representing the ultimate realization of personalized, data-driven medicine for CNO.

## 6. Conclusions

Charcot neuro-osteoarthropathy is a fundamentally biomechanical disease, driven by the interplay between a weakened skeleton and the mechanical forces of weight-bearing. For decades, its assessment and management have been hampered by a reliance on imaging modalities that provide only a static, unloaded view of the foot, leading to an incomplete and often misleading understanding of the deformity.

Weight-bearing CT has emerged as an important technological advancement that addresses this long-standing limitation. By providing the opportunity to visualize and quantify the 3D osseous architecture of the Charcot foot under true physiological load, WBCT has redefined the State-of-the-Art. This capability supports multiple stages of the management pathway, from enabling a more accurate and earlier diagnosis of instability and providing a quantitative basis for risk stratification to facilitating precision surgical planning and allowing for an objective evaluation of postoperative outcomes.

As technology becomes more accessible, the clinical community may increasingly consider integrating WBCT into the standard of care for the comprehensive assessment of CNO. Future research must focus on establishing standardized, validated 3D measurement protocols and conducting prospective studies to definitively link WBCT-derived metrics to long-term clinical outcomes. The combination of quantitative data from WBCT and the analytical capability of artificial intelligence holds the promise for supporting a shift toward more predictive and prescriptive approaches to CNO management from a reactive discipline to a predictive and ultimately prescriptive one, offering new hope for improving outcomes in this challenging patient population.

## Figures and Tables

**Table 2:** Key Clinical Applications of Weight-Bearing CT (WBCT) in Charcot Neuro-Osteoarthropathy.

Clinical Stage/Purpose	How WBCT Helps	Clinical Value
Early Diagnosis (Eichenholtz 0–1)	Detects subtle subluxation under load Reveals minimal joint incongruity Identifies occult instability invisible on MRI/non-WB CT [10,11]	Guides early offloading decisions Prevents progression to collapse Improves diagnostic accuracy when radiographs are normal [7]
Deformity Characterization	3D visualization of alignment in sagittal/coronal/axial planes [10] Quantifies lateral column height, FAO, impingement, and rotational deformity [12]	Enables objective severity assessment Provides risk markers for ulceration (e.g., lateral column collapse) [12]
Surgical Planning	Defines true load-bearing alignment Identifies instability sites and bony prominences Determines correction magnitude [13] Provides data for patient-specific instrumentation (PSI) [10]	More precise osteotomy/arthrodesis planning Better implant positioning Reduced operative uncertainty [13]
Postoperative Assessment	Evaluates correction under physiologic load Assesses fusion across arthrodesis sites [14] Detects recurrence, adjacent joint collapse, or loss of correction [13]	Objective confirmation of plantigrade alignment [14] Early identification of complications More accurate follow-up than radiographs [13]

**Table 3:** Complementary Roles of Weight-Bearing CT and MRI in Charcot Neuroosteoarthropathy.

Aspect	WBCT	MRI
Main strength	Load-dependent alignment and biomechanical instability	Early inflammation and marrow edema
Role across disease stages	Early: detection of latent instability under load Chronic: characterization of deformity	Early: identification of active inflammation All stages: soft tissue evaluation
Weight-bearing assessment	Yes	No
Bone alignment and collapse	Excellent	Limited
Soft tissue and marrow evaluation	Limited	Excellent
Role in clinical management	Guides offloading and surgical planning	Guides early diagnosis and activity assessment

## Data Availability

No new data were created or analyzed in this study.

## Abbreviations

The following abbreviations are used in this manuscript:

WBCT	Weight-Bearing Computed Tomography
CNO	Charcot neuro-osteoarthropathy
FAO	Foot and Ankle Offset
NWB	Non-weight bearing
TCC	Total contact cast
